# Virtually screening adults for depression, anxiety, and suicide risk using machine learning and language from an open-ended interview

**DOI:** 10.3389/fpsyt.2023.1143175

**Published:** 2023-06-12

**Authors:** Jennifer Wright-Berryman, Joshua Cohen, Allie Haq, David P. Black, James L. Pease

**Affiliations:** ^1^Department of Social Work, College of Allied Health Sciences, University of Cincinnati, Cincinnati, OH, United States; ^2^Clarigent Health, Mason, OH, United States

**Keywords:** suicide, depression, anxiety, machine learning, natural language processing, risk assessment, mental health, virtual screening

## Abstract

**Background:**

Current depression, anxiety, and suicide screening techniques rely on retrospective patient reported symptoms to standardized scales. A qualitative approach to screening combined with the innovation of natural language processing (NLP) and machine learning (ML) methods have shown promise to enhance person-centeredness while detecting depression, anxiety, and suicide risk from in-the-moment patient language derived from an open-ended brief interview.

**Objective:**

To evaluate the performance of NLP/ML models to identify depression, anxiety, and suicide risk from a single 5–10-min semi-structured interview with a large, national sample.

**Method:**

Two thousand four hundred sixteen interviews were conducted with 1,433 participants over a teleconference platform, with 861 (35.6%), 863 (35.7%), and 838 (34.7%) sessions screening positive for depression, anxiety, and suicide risk, respectively. Participants completed an interview over a teleconference platform to collect language about the participants’ feelings and emotional state. Logistic regression (LR), support vector machine (SVM), and extreme gradient boosting (XGB) models were trained for each condition using term frequency-inverse document frequency features from the participants’ language. Models were primarily evaluated with the area under the receiver operating characteristic curve (AUC).

**Results:**

The best discriminative ability was found when identifying depression with an SVM model (AUC = 0.77; 95% CI = 0.75–0.79), followed by anxiety with an LR model (AUC = 0.74; 95% CI = 0.72–0.76), and an SVM for suicide risk (AUC = 0.70; 95% CI = 0.68–0.72). Model performance was generally best with more severe depression, anxiety, or suicide risk. Performance improved when individuals with lifetime but no suicide risk in the past 3 months were considered controls.

**Conclusion:**

It is feasible to use a virtual platform to simultaneously screen for depression, anxiety, and suicide risk using a 5-to-10-min interview. The NLP/ML models performed with good discrimination in the identification of depression, anxiety, and suicide risk. Although the utility of suicide risk classification in clinical settings is still undetermined and suicide risk classification had the lowest performance, the result taken together with the qualitative responses from the interview can better inform clinical decision-making by providing additional drivers associated with suicide risk.

## 1. Introduction

Each year in the United States (US), more than 47,000 people die by suicide (1). Additionally, based on recent survey data from the US Census Bureau, 28.2% of adults endorsed symptoms of anxiety, 24.4% reported symptoms of depression, and 33.9% suffered from one or both conditions in the past 7 days (2). To address the growing rates of comorbid mental health conditions, there is a need for a singular, patient-centered, accurate, reliable, and objective tool to simultaneously identify patients at risk of suicide and other mental health disorders.

Universal screening tools deployed in a wide spectrum of facilities, including schools, physicians’ offices, outpatient, and inpatient facilities could address this problem, but the lack of a person-centered and objective tool along with a shortage of mental health clinicians in these settings is a major barrier to screening for coexisting depression, anxiety, and suicide risk on a public health scale. A common screening procedure involves filling out paper and pencil individual screeners for depression, anxiety, and suicide risk that are selected by the particular healthcare facility. Some common screeners include the nine-item Patient Health Questionnaire (PHQ-9), and the seven-item Generalized Anxiety Disorder (GAD-7), and the Columbia Suicide Severity Scale (C-SSRS), however, multiple others may be used, resulting in a lack of uniformity in scale and administration approach across settings. This method of screening does not allow for engagement between practitioner and patient, nor does it give space for a nuanced conversation about mental health, suicide risk and related patient needs. Also, even when screening instruments are part of a clinic’s protocol, they may not be consistently administered due to time constraints as separate instruments are required for each mental health condition (3). Further, these methods can be subject to self-report or clinician rating bias. Employing a brief, qualitative interview that collects the patient’s own words could fill a gap in current screening techniques by giving space for patients to discuss their needs ahead of crisis clinical decision-making.

Screening methods for depression, anxiety, and suicide risk have begun to shift in recent years as telehealth and other digital platforms have become increasingly prevalent. The trend towards using virtual methods for screening have gained momentum as the COVID-19 pandemic complicated in-person healthcare visits. Therefore, healthcare service users have become more aware of, and amenable to, accessing screening and treatment options virtually (4).

Natural language processing (NLP) and machine learning (ML) have expanded how mental health conditions can be identified (5, 6). Prior research from the Pestian lab undergirding the methods in this study used a corpus of suicide notes to train ML models (7, 8). From this, investigators developed an interview, called the Ubiquitous Questionnaire (UQ), to obtain language samples to further test the models in two clinical trials (9). The Adolescent Controlled Trial validated the ML model using the C-SSRS with 60 randomly selected emergency department cases (suicide complaints) and controls (orthopedic patients) (10). The model was able to correctly classify 97% of the participants as cases or controls. The second trial, Suicide Thought Markers (STM), randomly selected 379 adolescents and adults from mental health, suicide complaint, and control groups across three study sites. Results from the STM study indicated the model was able to identify the suicide group with 85% accuracy (11). Since the work in Pestian’s lab, innovations in NLP have demonstrated its screening efficiency and scalability in clinical and public health settings. Recent studies highlight the feasibility and clinical acceptability of using a digital platform to collect data through a 5-to-10-min interview for NLP analysis to identify suicide risk (12). Additionally, NLP models can perform well despite speakers’ varied location and regional dialect (13) making this method geographically portable (6).

Clairity, a depression, anxiety, and suicide risk screening program, uses NLP to identify all three conditions with a single brief interview. The purpose of this study was to (1) evaluate the feasibility of using a virtual platform to collect brief interviews for NLP analysis, and (2) to validate the ML models against the most widely used standardized instruments in a large, national sample. We also highlight the argument that a qualitative approach to screening is necessary to identify patient needs related to risk early in order to form a collaborative relationship and inform next steps in crisis and treatment planning. Given findings by Carter et al. (14, 15), guidance issued by the United Kingdom’s National Institute for Health and Care Excellence [NICE; (16)], and the call to action in determining how addressing patient needs is critical to preventing suicide, reducing risk, and improving quality of life, we propose that this method fills this gap by incorporating both a clinically-useful open-ended conversation and objective machine learning risk detection.

## 2. Methods

### 2.1. Study staff and participants

The study staff was composed of 18 clinical research coordinators (CRC). The CRCs completed online training to learn study procedures, principles of human subject protection, and good clinical practice. The CRCs oversaw all study procedures and were supervised by the clinical principal investigator.

**Figure 1:**
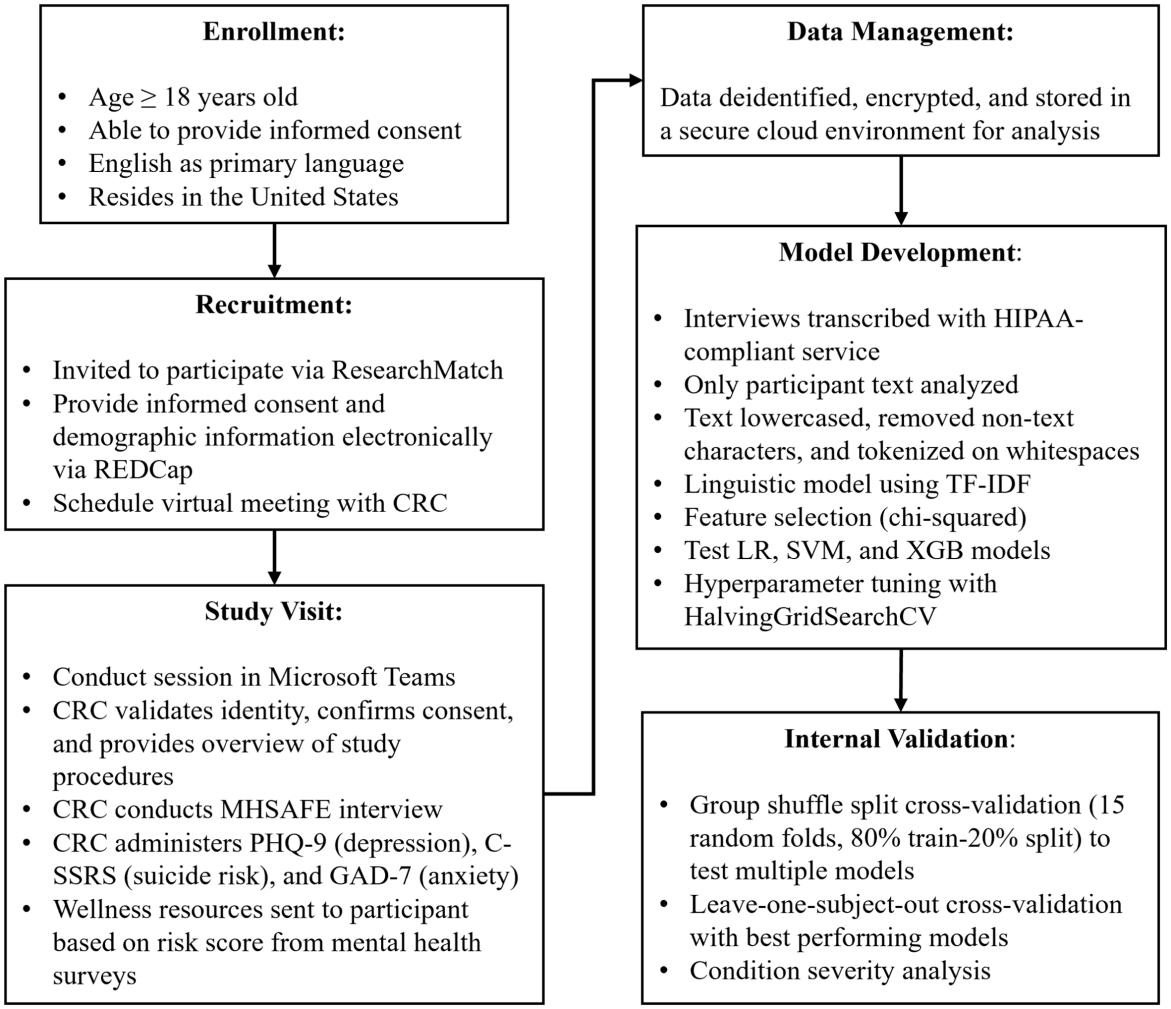
Schematic of study and modeling procedures.

Criteria for participant recruitment were: (1) age ≥18, (2) able to provide informed consent, and (3) English as a primary language. ResearchMatch (RM) was used to recruit for this study. RM is a national health volunteer registry created by several academic institutions and supported by the US National Institutes of Health as part of the Clinical Translational Science Award program. RM has an extensive pool of volunteers who have consented to be contacted by researchers about health studies for which they may be eligible. Approval for this study and all procedures was granted by a commercial Institutional Review Board. Participants received a $15 gift card for each session they completed (Figure 1).

Once matched, the participant completed the informed consent process, provided demographic information, and selected a session time via an online calendar system. Prior to the session, participants were sent reminders of their session by email and text. Microsoft Teams was used for all interviews.

### 2.2. Study design

Prior to the interview, participants’ identities were verified, and the CRC provided a brief overview of the study. Consent was confirmed and the CRC began the recording process. The CRC completed the 5-to-10-minute interview during which the CRC asked about the participant’s hopes, secrets, anger, fear, and emotional pain (MHSAFE). The MHSAFE interview is composed of standard prompts based on Pestian’s Ubiquitous Questionnaire, developed and tested to elicit emotional language for the screener (9–13, 17).

Survey data collected during the interview included the PHQ-9 (Patient Health Questionnaire-9 item), C-SSRS (Columbia-Suicide Severity Rating Scale) Screener, and GAD-7 (General Anxiety Disorder-7 item) for use in validation of the ML models and to produce a risk score. The resulting risk score prompted the CRC to follow the contingency and safety plan based on the participant’s identified risk level. Upon completion of the study, the participant was notified they may participate up to two more times. Participants scoring moderate or high risk were provided with resources including the 988 Suicide & Crisis Lifeline, the Crisis Text Line, and other tools such as the Stanley Brown Safety Plan (18). If the participant scored “high risk” on the mental health surveys, a more comprehensive contingency plan was followed, including asking additional questions about their mental state, access to lethal means, engagement in mental health services, and protective factors. In the event of imminent risk, the contingency safety plan included a warm hand-off to the 988 Suicide & Crisis Lifeline and/or a call to 911. To date, only one call to 911 was required during the study. This participant returned to complete additional interviews and was reported as safe.

Table 1 outlines the thresholds for each mental health condition. The PHQ-9 is a nine-item depression screener and is part of the full-length PHQ. The total score of the nine items ranges from 0 to 27, with a score of 10 used as a depression cut-off score. In a study conducted by Kroenke et al., a score of 10 or higher in the PHQ-9 had high sensitivity and specificity (88%) for detecting depression (19). The findings of this study were externally validated among different patient populations. The GAD-7 is a seven-item anxiety screener. In a reliability and validity study performed by Spitzer et al., various total cut-off points were analyzed for sensitivity, specificity, and validity. As the cut-off point increased, sensitivity decreases and specificity increases. However, at a total score of 10 or higher, sensitivity and specificity exceed 80% (20). Therefore, a score of 10 indicates a cut-off point for identifying anxiety cases.

**Table 1:** Assessments and case definitions.

Condition	Assessment	Case definition
Depression	PHQ-9	Total ≥ 10
Anxiety	GAD-7	Total ≥ 10
Suicide risk	C-SSRS	Risk ≥ Low

The C-SSRS Screener is a structured interview based on the full-length version (21). The first five questions measure suicidal ideation and behaviors in the past month on an ordinal scale. The last question measures suicidal behavior that occurred either in the past 3 months or have ever occurred over the lifetime The C-SSRS Screener designates a participant’s suicide risk level as “None” if all answers are negative, “Low” if there are non-specific suicidal ideations, “Moderate” if there is a method along with suicide ideation, or if there was lifetime suicidal behavior, “High” if there is active suicidal ideation with specific plan and intent, or if there was suicidal behavior within the past 3 months. For this study, a case is defined as someone who scores “Low” suicide risk or higher.

### 2.3. Data analysis

All analyses were performed using the Python programming language [version 3.9.12; (22)]. The open-source Python libraries Pandas [version 1.4.2; (23)], NumPy [version 1.22.3; (24, 25)], Scikitlearn [version 1.0.2; (26)], Matplotlib [version 3.5.1; (27)], and SciPy [version 1.8.0; (28)] were also used. Student’s *t*-tests were performed with SciPy’s *ttest_ind* function.

#### 2.3.1. Natural language processing and model development

The NLP/ML pipeline used in this study followed similar techniques used in previous work (10–13), focused on the term frequency-inverse document frequency (TF-IDF) of n-grams (contiguous sequence of n number of words). The text was preprocessed to be all lowercase and to remove any punctuation and non-letter characters. The text was tokenized with a simple whitespace tokenizer. Scikit-learn’s *SelectKBest* function was used for feature selection to identify features with the highest chi-square value. The ngram values (e.g., unigrams, bigrams, or trigrams) and the number of features selected were tunable hyperparameters.

We explored performance of three different models including logistic regression (LR), support vector machines (SVM), and extreme gradient boosting (XGB). LR is one of the simplest machine learning models yet still provides acceptable performance. SVMs have demonstrated excellent performance in previous tasks classifying suicidal language from semi-structured interviews, resist overfitting, and perform well in high-dimensional spaces (10–13). XGB has given state-of-the-art results on various problems and displayed promising results in a previous study (12, 29). Models were tuned using Scikit-learn’s *HalvingGridSearch* function with a stratified 5-fold cross-validation (CV) technique with non-overlapping subjects. Considered hyperparameters are available in Supplementary Table S1.

#### 2.3.2. Internal validation and performance evaluation

Initial model performance estimates were made using a group shuffle split (GSS) CV technique, where the dataset is broken into 15 randomly selected 80% train-20% test groups with non-overlapping subjects. We set the random state of the CV iterator to ensure consistent folds across experiments. This internal validation technique provides a more efficient estimate of model performance over a leave-one-subject-out (LOSO) CV technique. During model training, the only input was the participant’s language, labeled as case or control as defined in Table 1. During model testing, participant language was fed into the model and a probability for belonging to the case group was returned. Model performance was then determined by comparing the model predictions to the participant’s labeled group. At this stage, models were evaluated by the area under their receiver operating characteristic curves (AUC).

Models with the best GSS performance were then evaluated with a LOSO CV technique, where a model is iteratively trained on all but one subject’s sessions, and then makes a prediction on the held-out subject’s sessions. Because only one subject’s sessions are held out per CV fold, the model is the closest possible approximation to when it is trained on the full corpus. For results of LOSO CV, model performance was primarily evaluated with the AUC and Brier score. AUC values range from 0.5 (random chance) to 1.0 (perfect model). The Brier score is a measure of model calibration and ranges from 0 to 1 where a low score indicate less discrepancy between labels and predicted probabilities. Additional classification metrics calculated include accuracy, sensitivity, specificity, positive predictive value (ppv), and negative predictive value (npv). Thresholds for classification were determined as the maximum of sensitivity and specificity.

Feature weights were extracted for the best performing linear models. Feature weights for SVM models with a radial basis function (RBF), are not easily accessible, therefore the next best performing linear or tree-based model was used to identify important features. For linear models (LR or SVM with a linear kernel), feature weights are either positive or negative, indicating if they contribute to the model predicting a case or control, respectively.

#### 2.3.3. Model performance and condition severity

During model training, cases and controls were defined for each condition by accepted thresholds, shown in Table 1. Each instrument also has different severity levels for each condition based on the total scores. For the PHQ-9, severity increases for every 5 points of the total score, ranging from “None” (0–4), “Mild” (5–9), “Moderate” (10–14), “Moderately Severe” (15–19), and “Severe” (≥20). The GAD-7 follows the same severity levels, except there is no Moderately Severe bin, with scores ≥15 classified as “Severe.” For the C-SSRS, “None” results from negative answers to all questions; “Low” risk is characterized by passive suicidal ideation (SI); “Medium” risk by SI with methods or suicidal behavior longer than 3 months ago (lifetime); and “High” risk by suicidal intent with or without a plan, or suicidal behavior in the past 3 months.

Using the results from the LOSO CV for each condition, we computed model performance metrics for different severity levels. In this case the model was not retrained; the performance was computed by only selecting control sessions and the specified severity. For example, for the PHQ-9, “None” and “Mild” severities are considered controls, and the model’s performance for discriminating between “None” or “Mild” vs. “Moderate” depression was estimated by only considering sessions from the LOSO results where the severity is “None,” “Mild,” or “Moderate.”

## 3. Results

**Figure 2:**
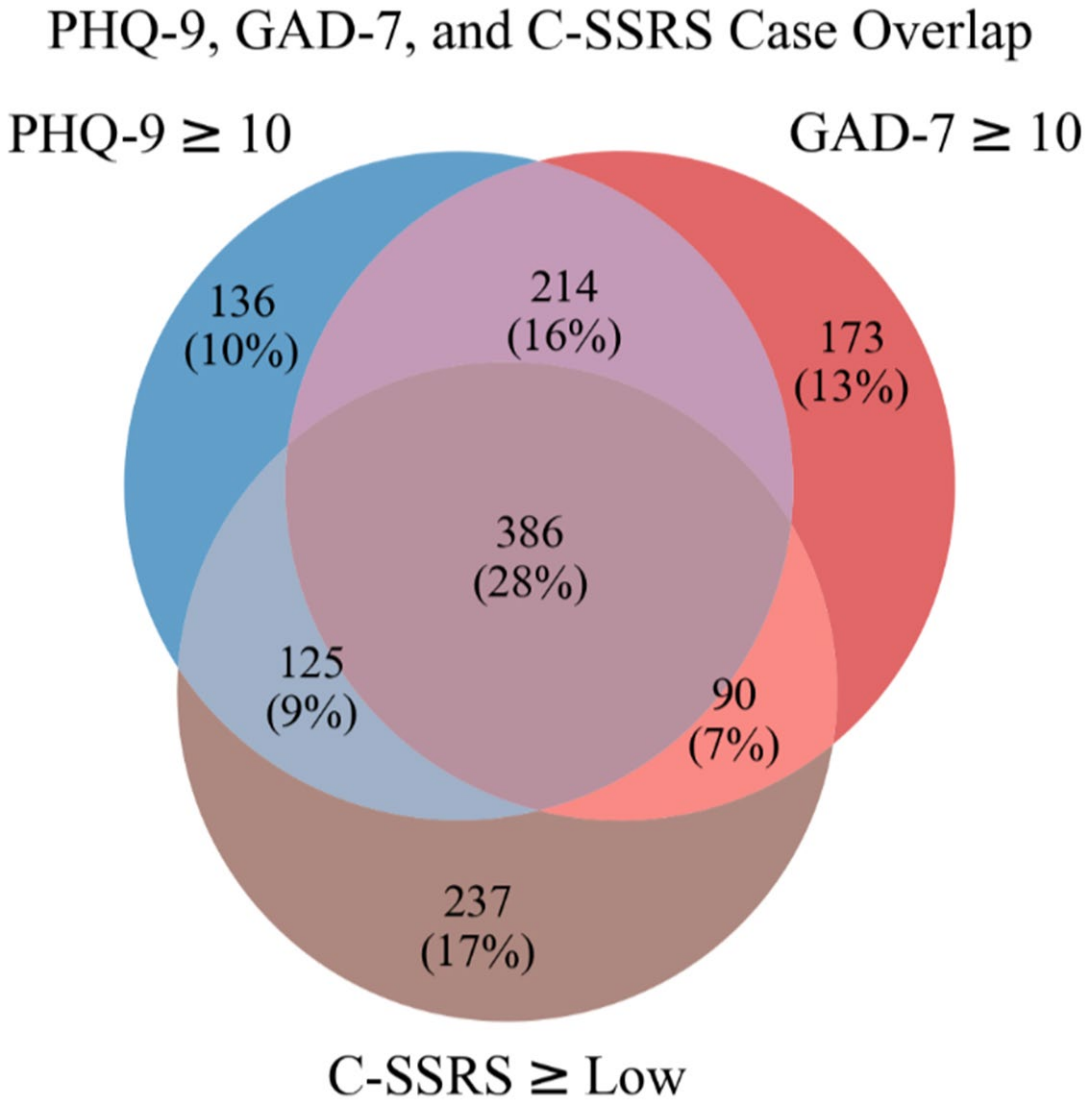
Venn diagram of case sessions.

Between November 2020 and August 2022, 1,433 participants were enrolled. Participants attended 1–3 sessions, which resulted in a total of 2,416 recorded sessions. The PHQ-9, GAD-7, and C-SSRS screeners were collected in all sessions. Participant demographics and the results of the mental health assessments are found in Table 2. Of the 2,416 sessions, 1,361 (56.3%) were classified as at least one case session. Figure 2 shows a Venn diagram of case session overlap. Most case sessions (28%) were positive for all three conditions, while those positive for both depression and anxiety had the next most overlap (16%). Those positive for only suicidal risk (17%) made up the largest number of sessions positive for a single condition.

**Table 2:** Participant descriptive statistics and case session summaries.

			Case sessions*		
	Participants	Sessions	PHQ-9 ≥ 10	GAD ≥10	CSSRS ≥ Low
Count (%)	1,433 (100%)	2,416 (100%)	861 (35.6%)	863 (35.7%)	838 (34.7%)
Average word count (SD)	–	856.9 (589.0)	830.5 (590.2)	856.7 (618.0)	854.1 (604.7)
Average interview length (min) (SD)	–	8.3 (4.6)	8.2 (4.4)	8.3 (4.9)	8.4 (4.7)
Average age (SD)	39.0 (13.8)	39.3 (14.8)	38.5 (13.5)	36.6 (12.7)	38.3 (14.2)
**Sex**					
Female (%)	1,132 (79.0%)	1922 (79.5%)	715 (29.6%)	724 (30.0%)	691 (28.6%)
Male (%)	284 (19.8%)	467 (19.3%)	131 (5.4%)	125 (5.2%)	130 (5.4%)
Prefer not to answer (%)	10 (0.7%)	17 (0.7%)	7 (0.3%)	5 (0.2%)	10 (0.4%)
Other (%)	5 (0.3%)	8 (0.3%)	8 (0.3%)	8 (0.3%)	5 (0.2%)
**Race**					
American Indian or Alaska Native (%)	14 (0.9%)	28 (1.2%)	12 (0.5%)	14 (0.6%)	10 (0.4%)
Asian (%)	84 (5.9%)	175 (7.2%)	50 (2.1%)	51 (2.1%)	62 (2.6%)
Black or African American (%)	126 (8.8%)	220 (9.1%)	80 (3.3%)	76 (3.1%)	66 (2.7%)
White or Caucasian (%)	1,146 (80.0%)	1,891 (78.3%)	678 (28.1%)	682 (28.2%)	658 (27.2%)
Native Hawaiian or Other Pacific Islander (%)	3 (0.2%)	5 (0.2%)	2 (0.1%)	1 (0.04%)	1 (0.04%)
Other (%)	58 (4.0%)	95 (3.9%)	39 (1.6%)	38 (1.6%)	40 (1.7%)
**Ethnicity**					
Non-Hispanic (%)	1,325 (92.5%)	2,245 (92.9%)	790 (32.7%)	784 (32.5%)	773 (32.0%)
Hispanic (%)	106 (7.4%)	169 (7.0%)	71 (2.9%)	78 (3.2%)	64 (2.6%)

*Percentages based on total number of sessions.

Demographic information was collected during the informed consent process. Participants were primarily female (79%, *N* = 1,132) and Caucasian (80%, *N* = 1,146). Other races represented in the sample were African American (8.8%, *N* = 126), Asian (5.9%, *N* = 84), Native Hawaiian or Other Pacific Islander (0.2%, *N* = 3) and Other (4%, *N* = 58). Over 7% of the sample reported a Hispanic ethnicity (7.4%, *N* = 106). The mean age of the sample was 39 ± 13.8 years. Of the 2,416 sessions, the average interview length was 8.3 min with a mean of 856.9 words per session. *t*-tests yielded no significant difference between case and control participants for interview length and word count for all mental health conditions.

### 3.1. Internal validation

#### 3.1.1. Group shuffle split

Table 3 displays AUCs and standard deviations (SD) across the GSS CV folds for each tuned model and condition. We found the best discrimination for the identification of depression (AUC = 0.76 ± 0.02) with LR and SVM (RBF kernel) models, using 2,048 and 1,024 features, respectively. We found good performance identifying anxiety (AUC = 0.74 ± 0.02) with LR and SVM (RBF kernel), with both models using 2048 features. Suicide had the lowest AUC of the three conditions (AUC = 0.70 ± 0.02), with an SVM (linear kernel) performing the best with 1,024 features. Before rounding, we found the SVM model performed slightly better for depression and suicide risk, while LR performed better for anxiety. Despite promising performance with XGB models in the past (12), XGB consistently had the lowest AUC. The optimal hyperparameters from the HalvingGridSearch for all the classifiers can be found in Supplementary Table S2.

**Table 3:** Model AUC per condition from GSS CV.

	Model AUC (SD)		
	LR	SVM	XGB
Depression	0.76 (0.02)	0.76 (0.02)	0.71 (0.02)
Anxiety	0.74 (0.02)	0.74 (0.02)	0.69 (0.02)
Suicide	0.70 (0.03)	0.70 (0.02)	0.66 (0.03)

#### 3.1.2. Leave-one-subject-out

Table 4 shows results of the LOSO CV for the best performing model during GSS CV for each condition. The performance estimates using GSS were consistent with these results, although we saw a 0.01 increase in AUC when identifying depression. The Brier scores ranged from 0.19 to 0.20 and the model score thresholds that optimized the sum of sensitivity and specificity ranged from 0.30 to 0.34.

**Table 4:** Model performance summary from LOSO results.

Condition	Depression	Anxiety	Suicide
Best model	SVM (rbf)	LR (liblinear)	SVM (linear)
AUC (95% CI)	0.77 (0.75–0.79)	0.74 (0.72–0.76)	0.70 (0.68–0.72)
Number of sessions	2,416	2,416	2,416
Number of cases	861	863	838
Brier score	0.19	0.20	0.20
Threshold	0.34	0.34	0.30
Sensitivity (95% CI)	0.72 (0.69–0.75)	0.57 (0.53–0.660)	0.70 (0.67–0.73)
Specificity (95% CI)	0.68 (0.65–0.70)	0.77 (0.75–0.79)	0.58 (0.55–0.60)
NPV (95% CI)	0.82 (0.79–0.84)	0.76 (0.74–0.78)	0.78 (0.76–0.81)
PPV (95% CI)	0.55 (0.53–0.58)	0.57 (0.54–0.61)	0.47 (0.44–0.50)
Accuracy (95% CI)	0.69 (0.68–0.71)	0.70 (0.68–0.71)	0.62 (0.60–0.64)

Table 5 shows the top 10 case and control features by feature weight for the best performing linear models fit to the entire dataset for each condition. Features from the LR model were used for depression because the feature weights from the better performing SVM with an RBF kernel were not readily accessible.

**Table 5:** Top 10 features for best performing models.

	Case features	Control features
Depression	cant, afraid, im, myself, get, just, mental, because, therapy, depression	no, think, good, we, about, well, new, say, so, worry
Anxiety	definitely, afraid, im, myself, because, therapy, anxiety, just, me, lot	no, think, not, guess, but, about, new, good, in, share
Suicide	its, feels, ive, want, yeah, suicidal, therapy, coping, mental, therapist	vaccinated, think, hold, would, no, hopeful, hopefully, school, you, now

#### 3.1.3. Condition severity

Table 6 shows classification performance metrics from the LOSO experiments broken out by severity level for each condition. In general, we saw a decrease in the number of case sessions and an increase in most model performance metrics as a condition’s severity increased. The only performance metric that did not improve with increasing severity is PPV, which is correlated with prevalence (30).

**Table 6:** Model performance for different condition severities.

	Depression			Anxiety		Suicide		
Severity	Mod.	Mod. Severe	Severe	Mod.	Severe	Low	Moderate	High
Sample size	2,071	1,793	1,662	2,069	1,900	1,896	2,025	1,651
Number of cases	516	238	107	516	347	318	447	73
AUC (95% CI)	0.72 (0.69–0.74)	0.80 (0.77–0.83)	0.92 (0.89–0.94)	0.69 (0.66–0.72)	0.81 (0.79–0.84)	0.70 (0.67–0.73)	0.67 (0.64–0.70)	0.85 (0.81–0.89)
Brier score	0.18	0.14	0.12	0.21	0.19	0.16	0.18	0.13
Threshold	0.27	0.33	0.44	0.44	0.53	0.35	0.27	0.44
Sensitivity (95% CI)	0.74 (0.70–0.78)	0.83 (0.77–0.87)	0.90 (0.83–0.94)	0.72 (0.67–0.75)	0.69 (0.64–0.74)	0.64 (0.59–0.70)	0.71 (0.67–0.75)	0.75 (0.64–0.84)
Specificity (95% CI)	0.60 (0.57–0.62)	0.66 (0.64–0.68)	0.78 (0.76–0.80)	0.55 (0.53–0.58)	0.77 (0.75–0.79)	0.64 (0.62–0.67)	0.53 (0.5–0.55)	0.77 (0.75–0.79)
NPV (95% CI)	0.87 (0.85–0.89)	0.96 (0.96–0.97)	0.99 (0.98–0.99)	0.85 (0.83–0.87)	0.92 (0.90–0.93)	0.90 (0.88–0.92)	0.87 (0.84–0.89)	0.99 (0.98–0.99)
PPV (95% CI)	0.38 (0.35–0.41)	0.27 (0.24–0.31)	0.22 (0.19–0.26)	0.35 (0.32–0.37)	0.40 (0.36–0.44)	0.27 (0.24–0.30)	0.30 (0.27–0.33)	0.13 (0.10–0.17)
Accuracy (95% CI)	0.63 (0.61–0.65)	0.68 (0.66–0.70)	0.79 (0.77–0.81)	0.59 (0.57–0.61)	0.75 (0.73–0.77)	0.64 (0.62–0.67)	0.57 (0.55–0.59)	0.77 (0.75–0.79)

We saw a slight decrease in model performance for moderate suicide risk compared to low suicide risk. This may be related to the lookback time of how the C-SSRS classifies moderate risk, where there are two paths to be classified as moderate risk: (1) suicidal ideation (SI) with a method in the past month and (2) suicidal behaviors >3 months ago (lifetime). Of the 447 moderate suicide risk sessions, only 84 (18.8%) answered positively to the SI with method question, while 267 sessions (59.7%) answered negatively to all C-SSRS questions except the lifetime suicidal behavior question. If the performance metrics from the LOSO results for moderate suicide risk are recomputed by considering only those with SI with a method as cases (84 sessions) and the 267 sessions whose suicidal risk was >3 months ago as additional controls, we find a 0.06 increase in AUC (AUC = 0.73; 95% CI = 0.67–0.79), indicating the model tends to score those whose risk was >3 months ago lower than those whose risk is more recent.

## 4. Discussion

The purposes of this study were to demonstrate feasibility of using a virtual platform to collect language data to screen for depression, anxiety, and suicide risk, and to validate machine learning models with a large and diverse national sample.

A recent meta-analysis of studies evaluating the use of technology to address mental health disorders found innovation in screening and mental health treatment has become widespread (31). Additionally, during the COVID-19 pandemic, telehealth and similar web-based methods were increasingly used for both research and treatment of mental disorders (32). However, to date, no single screening technique that simultaneously identifies depression, anxiety, and suicide risk has been developed or tested. Clairity is a program that analyzes linguistic features, collected via language samples, to identify all three disorders from a 5–10-min interview. We found using a virtual platform to conduct this study feasible, as we were able to recruit a large and diverse national sample of participants representing a range of people affected by depression, anxiety, and suicide risk, and comorbidities of the three disorders. This may be of particular importance to the medical and mental health field as the rise in prevalence of mental health disorders (33) warrants screening tools that are both portable and efficient.

Results from this study demonstrate both the efficiency and effectiveness of the Clairity program. First, the average interview time across participants was 8.3 min. In order to screen for these disorders using self-report standardized scales, patients in medical and mental health settings would fill out three separate scales, selecting responses that are the “best fit” for their current mental and suicide risk status. Time to complete these scales is one of the often-cited reasons why they are not completed consistently in settings such as primary care. Additionally, comorbidities (Figure 2) were identified using separate self-report standardized instruments. This type of screening procedure is inefficient and requires clinician scoring and interpretation of separate scales. A brief interview which identifies all three conditions simultaneously with a quick return of results may mitigate time constraints, patient (self)- or clinician-reporting bias, and clinician-nuanced scoring and lack of interpretation expertise (34–36). Lastly, a single interview also eliminates the “one size fits all” standardized screening response options.

The portability of the Clairity program allows for scalability as current mental health resources are stretched thin (37). Clairity is accessed via a web-based platform, allowing service users to complete the interview in various settings. For this study, participants were able to use their phones, desktop or laptop, or tablet, requiring an internet connection. CRCs were able to access results from the scales and follow-up with high-risk participants, providing resources including crisis lines and safety plans. In a clinic setting, service providers may elect to use Clairity in office, during a home visit, between visits, or a service user could access the program autonomously at home or in another private setting. Once the interview has been completed, results are then sent to the provider within seconds, with data from the three conditions being displayed in an accessible and intuitive dashboard.

The qualitative and machine learning output data available from the Clairity dashboard provides additional insights for clinicians when making collaborative decisions with patients about next steps in care. The language features (Table 5) related to depression, anxiety, and suicide risk offer clinical information not gleaned via standardized instruments in the form of thought markers. These thought markers (patient’s natural language) can aid in understanding the patient’s idiosyncratic risk identifiers. With further study of these features in a clinical setting, we can learn more about how these can be used in clinical decision-making and patient risk monitoring. Future studies will include how clinicians can use language features to inform risk levels and changes over time. Additionally, the information gathered through the qualitative interview will provide the clinician with patient-specific details about drivers and needs related to risk. Patient stories, of hope, anger, secrets, fears, and emotional pain can begin to paint the picture of the patient’s life experiences leading to their current mental state.

We found the best model performance identifying depression, followed by anxiety, with the poorest performance identifying any suicide risk. This is surprising considering the questions asked in the interview were originally developed with patients admitted to an emergency department for a suicide attempt or severe ideation, with model AUCs ranging from 0.69 to 0.93 depending on the features and CV method used. One possible explanation is that model performance, and consequently the separability of cases and controls, is related to condition severity. Indeed, Table 6 shows model performance tends to improve as the difference in condition severity between cases and controls increases for all conditions; one can imagine the classification task between “None” and “Severe” depression easier than between “Mild” and “Moderate” depression. This is noteworthy as the results from Table 6 did not include retraining the models, therefore the models did not have any explicit information about condition severity yet tended to rate more severe cases higher. In general, the PHQ-9, GAD-7, and C-SSRS Screener follow a linear progression, where more frequent or intense symptoms lead to higher severity classifications, except for the case of “Moderate” suicide risk, where this designation is still possible on the C-SSRS in the absence of any recent SI. Interestingly, this is where we observed the greatest discrepancy between the model and any mental health survey, and model performance improves when the language of those without any recent SI are considered controls. While the relationship between SI, lifetime suicidal behaviors, and risk designations is complex, the model evaluated in this study tends to rate those with lifetime suicidal behavior but no recent SI closer to controls. A person’s language with lifetime but no recent SI may accurately be classified as a control (low or no risk) by the model when their mental state reflects no *current or imminent* suicide risk thought or intention although the C-SSRS rates them as “Moderate” suicide risk.

Due to the low prevalence of suicide death, this form of classification of suicide risk has been met with scrutiny and evidence of utility in real-world clinical settings has been called into question by Carter et al. (14, 15). Given the modest predictive values of suicide risk screenings, Carter and colleagues warned that other ways to identify suicide risk are warranted. Although Clairity uses ML methods to provide data and a risk classification for suicide, the qualitative interview is intended to create a more useful path for communication between the patient and the interviewer. Where most, if not all, commonly used suicide screening techniques employ standardized scales, Clairity offers a new alternative to begin the conversation about risk, providing a patient-centered, non-confrontational exchange in an otherwise potentially volatile interaction. Additionally, Carter et al. suggest that modifiable factors, such as stressors or drivers of suicide, be assessed and addressed in treatment to reduce risk of suicide (14, 15). This is where Clairity’s “front door” approach may be particularly useful. By having an open-ended brief risk screening, respondents may disclose these suicide risk factors earlier, allowing for a seamless transition to critical next steps for safety and addressing issues related to suicide risk and treatment. However, additional exploration of how to offer a risk result is warranted, as the current classification system of “low-,” “moderate-,” and “high-” risk does not allow for the nuance of needs of individual patients (14, 15). The aim of the Clairity program is to disrupt this potentially ineffective system with a screening tool that provides critical information at the time of screening through qualitative responses.

Of the ML models tested during GSS CV, we found the tuned LR and SVM models to have similar performance. Surprisingly, XGB models had the poorest performance for all three conditions despite performing well in previous work and providing excellent results with other classification tasks (12, 29). It is possible additional hyperparameter tuning or feature scaling techniques may improve XGB’s classification performance, although it may also be XGB models are not ideal for classification tasks with the high dimensional, sparse matrices used in this study.

During LOSO CV, we found agreement with the GSS CV results, indicating GSS CV was a reasonable method to estimate model performance on the entire dataset. The classification thresholds for the calculation of the additional performance metrics shown in Table 4 were determined as the value that maximized the sum of sensitivity and specificity. Coincidentally, these thresholds are within a few percentage points of the percentage of case sessions for each condition for this slightly imbalanced dataset. There are many methods to determine a classification threshold, and the selection of one will depend on the specific clinical context and an appropriate balance between the cost of false positives and false negatives of the classification task. A recent analysis by Ross et al. examined accuracy requirements for cost-effective suicide risk prediction in US primary care patients and found the two interventions examined – active contact and follow-up (ACF) and cognitive behavioral therapy (CBT) – cost-effective, provided the model performed with a specificity of 95.0% and a sensitivity of 17.0% for ACF and 35.7% for CBT in predicting a suicide *attempt* (38).

Our reference instruments report sensitivity and specificity values exceeding 80%, while our models’ performance estimates are lower, as observed in Table 4. However, caution is necessary when drawing comparisons based solely on performance estimates. All performance metrics, whether applied to an ML model or traditional instrument, reflect estimations of the expected performance of the tool in real-world contexts. Thus, it is important to consider the method by which the performance metrics were calculated before making any comparisons. For instance, the initial validation study of the PHQ-9 by Kroenke et al. involved mental health professionals interviewing 580 primary care patients, of whom 41 (7%) were identified as having major depressive disorder (MDD) (19). These cases formed the basis for the sensitivity and specificity estimates of the PHQ-9. Similarly, we found that our models’ performance improved as the severity of depression and the likelihood of MDD increased. Furthermore, the classification of depression is complicated by the “gray zone” (a range of depression severity not easily classified as either case or control), which poses a challenge for both the PHQ-9 and our models (19). Thus, the classification task is more challenging when patients fall within this gray zone. Lastly, while our models used a relatively large sample size, accurately representing the complexity and nuance of language remains a challenge. The TF-IDF approach we used in our study provides a simplified representation of language. Thus, more advanced NLP techniques may improve model performance by better accounting for the intricacies of natural language.

Table 5 shows the top 10 case and control features by feature weight for the best performing linear models for each condition. While these features represent a small fraction of the total number of features and a full linguistic analysis is beyond the scope of this paper, some interesting patterns emerge. For each condition analyzed, the condition itself appears as a top feature, and for all models, the word “therapy” was a top case feature. Other work has found increased personal pronoun usage related to depression and suicide risk (39), often interpreted as more inwardly-focused. We found the pronouns “im,” “me,” “myself,” and “ive” as part of the top 10 case features across the three models. We found the top features had the greatest overlap between the depression and anxiety models, especially for the control features, which is congruent with the larger overlap of case sessions for these conditions shown in Figure 2. For the suicide risk model, the word “vaccinated” was the top control feature, likely related to the start of the study coinciding with the national COVID-19 vaccination effort.

### 4.1. Limitations and future directions

While these findings agree with previous studies, some limitations should be noted. First, while the PHQ-9, GAD-7, and C-SSRS screeners have reported acceptable validity, recent literature has highlighted the inadequacy of screeners to accurately identify those at risk at the time of screening (35, 40). These screeners were used as our source of ground truth for model development and validation. Thus, any inaccuracies in our ground truth labels would impact our models’ performance estimates. The thresholds used in Table 1 to identify each condition were selected to maximize the instrument’s sensitivity and specificity, therefore we anticipate any mislabeling to be roughly equal for cases and controls, and the models’ true performance to remain within the reported confidence intervals.

Suicide risk is comprised of numerous factors, including personal, environmental, and time (41–44). Therefore, screening methods should be used as an opportunity to not only identify the presence of risk, but also to begin building a safe space for patients to discuss needs related to risk. The use of brief quantitative screeners cannot be relied upon to engage the patients on their needs or the complicated array of contributors to imminent risk. More comprehensive needs assessments, which are the step after screening is implemented, could help explain discrepancies between our model and the C-SSRS, such as our model’s tendency to rate individuals with lifetime but no recent SI closer to controls. Additionally, it should be noted that screening tools, such as Clairity, are not intended to fully assess risk and patient needs for determining the plan of care. In light of the work of Carter et al. (14, 15), it is important that the needs of the individual as assessed during comprehensive and narrative interviewing post-screening should form a collaborative and patient-centered treatment course. If suicide risk, depression, and anxiety screenings are used to decide course of action, patients might be subjected to traumatizing and expensive treatment that does not address their specific needs better assessed by exploring the conditions underlying the risk.

There may be some limitations for users when technology is employed to gather screening data. First, the participant or client must have access to a device and the internet. This may be a challenge for some who have limited access to either of these. However, this challenge can be offset if the provider uses Clairity in office. Second, internet instability, noise in the room, or a participant’s style of speaking can alter the language sample. For this study, six sessions (0.2%) were eliminated due to these issues. Some users may find a telehealth approach less patient-friendly and personal, however, many patients report benefit of virtual options in terms of accessibility (45). Last, some clinics’ adoption of new technology may be slow, even when clinical users accept new and innovative methods of care (46).

Additional limitations include the use of recruited research volunteers who were incentivized to answer the questions in the MHSAFE interview. Patients in clinical settings in which the provider is conducting the interview may respond differently. This could affect the generalizability of the models when applied in other settings. Additional external validation studies could help to identify the extent of this limitation. In a previous emergency department study, we solicited feedback from clinician users of the MHSAFE interview and gleaned their perception of differences in patient risk disclosure when comparing the C-SSRS to Clairity. Clinicians stated the interview was more patient-centered, and patients were more forthcoming during the open-ended approach. They also reported some patients felt “what they were saying was important” and they felt “seen and heard” (47).

Future work will examine both the use of qualitative data in collaborative clinical decision-making related to patient needs and model performance across different participant demographics and settings (e.g., emergency departments and outpatient therapy), how features can be used to identify patterns in thought markers related to risk, and a repeated measures analysis. We also plan to investigate the use of more advanced NLP techniques that leverage large language models and may account for a more linguistic nuance but may also retain biases (48–51). Lastly, we want to explore how the type of return of results meaningfully informs clinical decision-making whereby the qualitative data from the interview are used in the context of the presence of suicide risk to identify what the individuals needs are related to reducing suicide risk and improving wellbeing.

## 5. Conclusions

The results of this large, national study of the use of a virtual platform to conduct mental health and suicide risk screening suggests it is feasible to simultaneously identify depression, anxiety, and suicide risk from a brief qualitative interview. The methods utilized in this study were modified from those used in outpatient therapy and emergency departments, and they might be easily applied to other settings where early detection may improve outcomes. Although suicide risk classification is still tentative in its utility as suggested in the literature and its lower relative model performance, the MHSAFE interview can offer additional insights about risk factors and related patient needs. The qualitative data along with the risk classification can support clinical decisions and set meaningful next steps in motion. Future work will include a randomized controlled trial to study performance in mental health settings with clinical outcomes.

## Data availability statement

The original contributions presented in the study are included in the article/Supplementary material, further inquiries can be directed to the corresponding author.

## Ethics statement

The studies involving human participants were reviewed and approved by Advarra. The patients/participants provided their written informed consent to participate in this study.

## Author contributions

JW-B, JC, and JP wrote the manuscript. JC, AH, and DB performed statistical analysis on the corpus. JC and JW-B are principal investigators of the study Classification and Assessment of Mental Health Performance Using Semantics—Expanded. All authors contributed to the article and approved the submitted version.

## Funding

The authors declare that this study received funding from Clarigent Health. The funder was not involved in the study design, collection, analysis, interpretation of data, the writing of this article, or the decision to submit it for publication.

## Conflict of interest

JW-B and JP are part-time consultants and are sponsored by a grant from Clarigent Health. JC, DB, and AH are employed full-time by Clarigent Health. The above interests do not alter our adherence to Frontiers Media’s policies. Clarigent Health did not influence or restrict the submission of this publication.

## Publisher’s note

All claims expressed in this article are solely those of the authors and do not necessarily represent those of their affiliated organizations, or those of the publisher, the editors and the reviewers. Any product that may be evaluated in this article, or claim that may be made by its manufacturer, is not guaranteed or endorsed by the publisher.

## Glossary

**Table:** 

AUC	Area under receiver operating characteristic curve
C-SSRS	Columbia-suicide severity rating scale
CI	Confidence interval
CRC	Clinical research coordinator
CV	Cross-validation
GAD-7	Generalized anxiety disorder 7-item
GSS	Group-shuffle-split
LOSO	Leave-one-subject-out
LR	Logistic regression
MHSAFE	Mental health hopes secrets anger fear and emotional pain
ML	Machine learning
NLP	Natural language processing
NPV	Negative predictive value
PHQ-9	Patient health questionnaire 9-item
PPV	Positive predictive value
RM	Research match
SD	Standard deviation
STM	Suicide thought markers (study)
SVM	Support vector machine
XGB	Extreme gradient boosting
